# Prevalence of zonulopathy in primary angle closure disease

**DOI:** 10.1111/ceo.13983

**Published:** 2021-08-31

**Authors:** Ali Salimi, Anthony Fanous, Harrison Watt, Mohamed Abu‐Nada, Anna Wang, Paul Harasymowycz

**Affiliations:** ^1^ Department of Ophthalmology, Faculty of Medicine McGill University Montreal Quebec Canada; ^2^ Montreal Glaucoma Institute and Bellevue Ophthalmology Clinics Montreal Quebec Canada; ^3^ Faculty of Medicine McGill University Montreal Quebec Canada; ^4^ Department of Ophthalmology University of Montreal Montreal Quebec Canada

**Keywords:** primary angle closure disease, capsular tension ring, cataract surgery, pigment dispersion syndrome, zonulopathy

## Abstract

**Background:**

To determine the prevalence of zonulopathy in a large cohort of eyes with primary angle closure disease (PACD) that underwent cataract surgery.

**Methods:**

Retrospective consecutive case series of PACD eyes (including primary angle closure suspect, primary angle closure, and primary angle closure glaucoma) that underwent phacoemulsification cataract surgery or clear lens extraction between 2009 and 2020 at a single ophthalmology centre. Those with risk factors for zonulopathy such as history of trauma, pseudoexfoliation syndrome, intraocular surgery, retinitis pigmentosa or connective tissue disorders were excluded. The primary outcomes included the prevalence of zonulopathy assessed intraoperatively and secondary pigment dispersion syndrome.

**Results:**

In our cohort of 806 consecutive PACD eyes, the prevalence of zonulopathy was 7.3% (59 of 806 eyes) – significantly greater than the 0.46%–2.6% range reported for the general population (*p* < 0.001). Intraoperative signs of zonular weakness included floppy capsular bag (29 eyes, 3.6%), zonular laxity (25 eyes, 3.1%) and zonular dehiscence (11 eyes, 1.4%). Among these eyes, capsular tension ring was used in 23 eyes (39.0%), six eyes (10.2%) experienced vitreous prolapse intraoperatively and underwent anterior vitrectomy, and two eyes (3.4%) experienced posterior capsular rupture, one of which required a scleral‐fixated intraocular lens. Secondary pigment dispersion syndrome was observed in 141 eyes (17.5%).

**Conclusions:**

This study evidenced a high prevalence of zonulopathy among a large cohort of PACD eyes and suggests zonulopathy as a possible under‐recognised cause of angle closure. Until more sophisticated imaging modalities become available, awareness about the prevalence of zonulopathy in angle closure disease coupled with careful preoperative examinations can help minimise or prevent the complications of zonulopathy.

## 1 INTRODUCTION

Primary angle closure disease (PACD) includes primary angle closure suspect (PACS), primary angle closure (PAC), and primary angle closure glaucoma (PACG) ‐ a spectrum of conditions from normal to progressive optic neuropathy that have appositional or synechial closure of the anterior chamber angle.^1^ While the majority of glaucoma cases are open‐angle, PACG has a much more acute and rapidly progressive disease course which puts this condition at a higher risk of permanent neuronal damage and vision loss.^2^ In East Asia, PACG is the leading cause of irreversible blindness and is projected to affect 32 million adults aged between 40 and 80 years old by 2040.^3^ PACD is multifactorial with a variety of risk factors including increasing age; female sex; Asian, Inuit, Mongolian and African ethnicity; shallow anterior chamber depth; and hyperopia.^4^, ^5^, ^6^

Laser peripheral iridotomy (LPI) has traditionally been the mainstay in the prophylaxis or treatment of PACD, as it can eliminate the pupillary block and allow widening of the iridocorneal angle.^7^ However, its efficacy is variable and a large proportion of patients (up to 81.8%) may still have gonioscopic angle closure after a year.^8^ More recently, the evidence from the EAGLE study supporting the efficacy and cost‐effectiveness of clear lens extraction (CLE) in PAC and PACG has led to a paradigm shift toward early cataract surgery or CLE as a treatment option for PACD.^9^

Cataract surgery is one of the most commonly performed elective surgeries with an excellent safety profile. Nonetheless, it still bears the risk of intraoperative and postoperative complications. Zonulopathy is a clinical diagnosis that presents with inadequate zonular support for the lenticular capsule.^10^ Zonular weakness has been associated with several intraoperative complications and postoperative adverse outcomes including vitreous prolapse, capsular rupture or contraction, intraocular lens (IOL) decentration or dislocation, and pseudophacodonesis.^10^, ^11^, ^12^ The common risk factors for zonulopathy include pseudoexfoliation syndrome, ocular trauma, iatrogenic zonulysis, connective tissue disorders, retinitis pigmentosa, and aniridia.^10^, ^13^, ^14^ Early detection of zonulopathy can help prepare for surgical challenges in this population. The preoperative signs include phacodonesis, lens tilting or subluxation, extremely shallow or deep anterior chamber depth, asymmetry in the anterior chamber depth or axial length compared to the contralateral eye, and presence of an iridolenticular gap.^12^, ^15^ Secondary pigment dispersion syndrome (SPDS) could also be an indirect sign of zonulopathy, as zonular weakness could lead to forward movement of the lens which in turn can cause iris chaffing from an IOL.

To the best of our knowledge, the prevalence of zonulopathy in PACD has not been previously reported. With the recent increase in popularity of CLE in the management of PAC and PACG^16^ and the rather asymptomatic nature of zonulopathy during the preoperative period,^17^ it is all the more important to study the prevalence of this pathology in eyes with PACD. Thus, the present study evaluates the prevalence of zonulopathy and SPDS in a large cohort of PACD eyes, in the hope of early detection of such cases and potentially minimising the associated complications.

## 2 METHODS

### 2.1 Study design and population

This retrospective case series consisted of 806 consecutive PACD eyes (including PACS, PAC, and PACG) that underwent phacoemulsification cataract surgery or CLE with or without concomitant glaucoma surgeries, by a single surgeon, at the Hôpital Maisonneuve Rosemont (Montreal, Canada), between January 2009 and August 2020. Preoperatively, indentation gonioscopy was performed at the slit lamp with minimal illumination in order to avoid pupillary constriction. Patients were classified as PACS, PAC or PACG, according to the angle closure staging classification set by the American Academy of Ophthalmology Primary Angle Closure Preferred Practice Pattern® guidelines^1^: PACS was defined as iridotrabecular contact for greater than 180° in absence of peripheral anterior synechiae (PAS), elevated intraocular pressure (IOP), or optic neuropathy; PAC was defined as PACS with IOP over 21 mmHg or PAS, without evidence of optic neuropathy; and PACG was defined as PAC with glaucomatous optic neuropathy. Patients with known risk factors for zonulopathy including history of trauma, pseudoexfoliation syndrome, previous intraocular surgery, retinitis pigmentosa, or connective tissue disorders were excluded.

The study was approved by the board of ethics of the Hôpital Maisonneuve‐Rosemont (Montreal, Canada) and was conducted in accordance with the 1964 Declaration of Helsinki and its later amendments or comparable ethical standards. All patients signed voluntary informed consent.

### 2.2 Surgical technique

All procedures were performed by the same surgeon. Under sterile conditions and after the instillation of topical anaesthesia, a temporal clear corneal incision was performed. Standard phacoemulsification cataract surgery or CLE was followed, and a foldable IOL was placed in the capsular bag. Finally, the viscoelastic was exchanged with balanced salt solution, the incision site was hydrated, and water tightness was ensured. In cases with extensive PAS, prior to phacoemulsification, goniosynechialysis (GSL) was performed by pressing a blunt cyclodialysis spatula against the peripheral edges of the iris next to areas with angle adhesion. In cases with evidence of zonular deficiency or weakness, a capsular tension ring (CTR) was used to stabilise and centre the capsule. PACG eyes with uncontrolled IOP or evidence of disease progression underwent the standard phacoemulsification cataract surgery or CLE as described above with a concomitant glaucoma procedure.

The standard postoperative regimen included topical moxifloxacin 0.5% (three times a day for 1 week), topical nepafenac ophthalmic solution 0.1% (three times a day for 1 month), and loteprednol etabonate 0.5% (four times a day tapered down over 1 month, except for cases of iStent where they were tapered within 2 weeks). For PAC and PACG patients that were preoperatively on anti‐glaucoma medications, the postoperative dose was adjusted on a case‐by‐case basis, at the surgeon's discretion according to the preoperative IOP, disease severity, tolerance of the eye drops, and the desired target IOP.

### 2.3 Outcome measures

Baseline demographics, ocular characteristics, as well as intraoperative and postoperative data of eyes, were reviewed and extracted from patients' medical records. The primary outcome measures were the prevalence of zonulopathy as detected intraoperatively, and preoperative evidence of SPDS. Zonulopathy signs included one or several of the following intraoperative signs with or without the use of a CTR: evidence of phacodonesis; lens tilting or subluxation; wrinkling of the anterior capsule during manual capsulorhexis; loose or floppy capsular bag, infolding of peripheral capsule or visualisation of the capsular equator during the cortical or nuclear removal; zonular dehiscence; zonular laxity; presence of a large iridolenticular gap; and a varying shallow and deep anterior chamber during irrigation. SPDS was defined as the presence of three out of the following four signs: loss of pupillary sphincter; presence of pigment granules on anterior iris stroma; endothelial cell pigmentation deviant from the classic Krukenberg's spindle pattern; and increased angle pigmentation. Secondary outcome measures included best‐corrected visual acuity (BCVA) at the first postoperative month, the association between zonulopathy and the baseline characteristics, as well as safety measures including intraoperative use of CTR, intraoperative complications, and postoperative adverse events. IOP spike was defined as IOP greater than 10 mmHg or 50% increased relative to baseline.^18,19^ All ocular biometric measures were obtained using LenStar LS 900 optical biometer (Haag Streit AG, Switzerland).

### 2.4 Statistical analysis

The prevalence of zonulopathy in our cohort was determined and compared between PACS, PAC and PACG groups using Fisher's exact test. The same test contrasted the zonulopathy rate in our cohort with those previously reported for the general population. Univariate and multivariate binary logistics regression models assessed the baseline characteristics associated with zonulopathy. Postoperative change in BCVA was examined using repeated‐measure ANOVA. Statistical analyses were performed using the SPSS (version 26, IBM, USA) and were corrected for correlation between eyes of the same patient with alpha level set at 0.05 for statistical significance.

## 3 RESULTS

A total of 806 eyes of 465 patients were included. The cohort consisted of 36% males and 64% females with an average age of 65.70 ± 10.70 years. PACD diagnoses included PACS in 170 eyes (21%), PAC in 349 eyes (43%) and PACG in 287 eyes (36%). Prior glaucoma interventions included LPI in 80%, Argon laser trabeculoplasty in 12%, and selective laser trabeculoplasty in 5% of the eyes, while no eye had a history of incisional ocular surgery prior to phacoemulsification. Preoperatively, the average axial length was 22.54 ± 1.16 mm, anterior chamber depth was 2.70 ± 0.33 mm, and lens thickness was 4.72 ± 0.52 mm. Phacoemulsification cataract surgery or CLE was performed as a stand‐alone procedure in 505 eyes (63%) and in combination with a glaucoma surgery in the remaining 301 eyes (37%), including iStent or iStent *inject* trabecular micro‐bypass stent (194 eyes), endoscopic cyclophotocoagulation (37 eyes), Ex‐PRESS shunt drainage device (22 eyes), XEN gel stent (14 eyes), non‐penetrating glaucoma surgery (12 eyes), Trabectome (9 eyes), Hydrus micro‐stent (5 eyes), trabeculectomy (5 eyes) and Cy‐Pass micro‐stent (3 eyes). GSL was performed in 36% of the eyes. Preoperatively, average intraocular pressure was 15.83 ± 4.83 mmHg and the mean number of glaucoma medications used was 1.40 ± 1.49 medications. Baseline demographics and ocular characteristics for the whole cohort and sub‐groups stratified by angle closure diagnosis are presented in Table 1.

**TABLE 1:** Demographics and baseline ocular characteristics

Variable	Whole‐Cohort *N* = 806	PACS *N* = 170	PAC *N* = 349	PACG *N* = 287
Age (years)	65.70 ± 10.70	67.01 ± 9.92	62.84 ± 10.15	68.40 ± 10.96
Sex (Male : Female); *n* (%)	287 (36%) : 519 (64%)	45 (26%) : 125 (74%)	112 (32%) : 237 (68%)	130 (45%) : 157 (55%)
Eye (OD : OS); *n* (%)	413 (51%) : 393 (49%)	87 (51%) : 83 (49%)	178 (51%) : 171 (49%)	148 (52%) : 139 (48%)
Diabetes; *n* (%)	66 (8%)	12 (7%)	22 (6%)	32 (11%)
Prior glaucoma interventions; *n* (%)				
Laser peripheral iridotomy	648 (80%)	141 (83%)	291 (83%)	216 (75%)
Argon laser trabeculoplasty	100 (12%)	15 (9%)	62 (18%)	23 (8%)
Selective laser trabeculoplasty	38 (5%)	0 (0%)	8 (2%)	30 (11%)
Surgery combined with GSL; *n* (%)	294 (36%)	0 (0%)	170 (49%)	124 (43%)
Central corneal thickness (μm)	556.24 ± 40.14	553.26 ± 38.44	561.95 ± 39.85	551.16 ± 40.72
Intraocular pressure (mmHg)	15.83 ± 4.83	14.19 ± 2.77	16.23 ± 4.74	16.33 ± 5.62
Glaucoma medications	1.40 ± 1.49	0.00 ± 0.00	1.09 ± 1.25	2.35 ± 1.43
Best corrected visual acuity (logMar)	0.27 ± 0.51	0.23 ± 0.36	0.21 ± 0.42	0.37 ± 0.66
Cup to disc ratio	0.55 ± 0.23	0.48 ± 0.18	0.46 ± 0.20	0.70 ± 0.21
Axial length (mm)	22.54 ± 1.16	22.34 ± 1.09	22.32 ± 1.20	22.93 ± 1.06
Anterior chamber depth (mm)	2.70 ± 0.33	2.69 ± 0.28	2.71 ± 0.33	2.69 ± 0.36
Lens thickness (mm)	4.72 ± 0.52	4.83 ± 0.44	4.72 ± 0.51	4.65 ± 0.55
Glaucoma Severity; *n* (%)				
Mild	111 (14%)	–	–	111 (39%)
Moderate	82 (10%)	–	–	82 (29%)
Severe	94 (12%)	–	–	94 (33%)

*Note*: Mean ± Standard deviations are presented, where applicable. GSL: Goniosynechialysis; PACS: primary angle closure suspect; PAC: primary angle closure; PACG: primary angle closure glaucoma.

Table 2 illustrates the prevalence of zonulopathy in our cohort. A total of 59 eyes were found to have zonulopathy – representing 7.3% of the cohort. Among these eyes, 29 had a floppy capsular bag (3.6% of the cohort), 25 eyes had zonular laxity (3.1% of the cohort), and 11 eyes had zonular dehiscence (1.4% of the cohort). The prevalence of zonulopathy in the general population ranges between 0.46% and 2.6%.^20^, ^21^, ^22^, ^23^ Comparing the higher end of this range (2.6%, reported by Girgis et al.^23^) to our cohort, we evidenced a significantly higher prevalence of zonulopathy in PACD eyes (*p* < 0.001). SPDS was observed among 141 eyes (17.5%; Table 2). Stratification by angle closure diagnosis did not reveal any significant intergroup differences with respect to the zonulopathy rate (*p* > 0.05).

**TABLE 2:** Prevalence of zonulopathy and secondary pigment dispersion syndrome in the whole cohort and stratified by angle closure diagnosis

Variable	Whole‐Cohort *N* = 806	PACS *N* = 170	PAC *N* = 349	PACG *N* = 287	*p*‐Value
Intraoperative evidence of zonulopathy; *n* (%)	59 (7.3%)	12 (7.1%)	23 (6.6%)	24 (8.4%)	0.905
Floppy capsular bag; *n* (%)	29 (3.6%)	6 (3.5%)	9 (2.6%)	14 (4.9%)	0.280
Zonular laxity; *n* (%)	25 (3.1%)	3 (1.8%)	12 (3.4%)	10 (3.5%)	0.780
Zonular dehiscence; *n* (%)	11 (1.4%)	3 (1.8%)	4 (1.1%)	4 (1.4%)	0.324
Secondary pigment dispersion syndrome; *n* (%)	141 (17.5%)	35 (20.6%)	50 (14.3%)	56 (19.5%)	0.200

Abbreviations: PACS: primary angle closure suspect; PAC: primary angle closure; PACG: primary angle closure glaucoma.

Table 3 highlights the association between baseline ocular characteristics and zonulopathy. The univariate model evidenced an association between zonulopathy and male sex (*p* = 0.035), diabetes (*p* = 0.047), worse baseline BCVA (*p* = 0.021), and smaller anterior chamber depth (*p* = 0.008). The multivariate model including these four variables highlighted an association between zonulopathy and diabetes (*p* = 0.045) and smaller anterior chamber depth (*p* = 0.009). Other factors such as age, IOP, axial length, prior glaucoma interventions, and the PACD subtypes were not associated with zonulopathy (*p* > 0.05).

**TABLE 3:** Binary logistics regression of association between baseline ocular characteristics and zonulopathy

	Univariate			Multivariate		
Variable	B	95% CI	*p*‐Value	B	95% CI	*p*‐Value
Age (years)	1.003	0.978–1.028	0.843			
Sex			0.035^a^			0.191
Female (reference)	–	–	–	–	–	–
Male	1.782	1.041–3.049	0.035^a^	1.474	0.824–2.637	0.191
Eye			0.850			
OD (reference)	–	–	–			
OS	0.950	0.558–1.616	0.850			
Diabetes	2.179	1.008–4.500	0.047^a^	2.241	1.018–4.930	0.045^a^
Prior laser peripheral iridotomy	1.116	0.563–2.211	0.754			
Prior Argon laser trabeculoplasty	0.658	0.256–1.690	0.384			
Prior selective laser trabeculoplasty	1.601	0.546–4.699	0.391			
Surgery combined with GSL	0.960	0.552–1.671	0.886			
Central corneal thickness (μm)	1.005	0.999–1.012	0.118			
Intraocular pressure (mmHg)	1.027	0.977–1.079	0.291			
Glaucoma medications	0.946	0.781–1.145	0.567			
Best corrected visual acuity (logMar)	1.566	1.069–2.296	0.021^a^	1.207	0.727–2.007	0.469
Cup to disc ratio	1.337	0.383–4.671	0.649			
Axial length (mm)	0.832	0.635–1.015	0.064			
Anterior chamber depth (mm)	0.306	0.127–0.739	0.008^a^	0.314	0.131–0.750	0.009^a^
Lens thickness (mm)	2.008	0.946–4.262	0.070			
Angle closure classification			0.904			
Primary angle closure suspect (reference)	–	–	–			
Primary angle closure	0.872	0.443–1.715	0.869			
Primary angle closure glaucoma	0.980	0.497–1.930	0.953			

^a^ Denotes statistical significance. GSL: Goniosynechialysis.

Average BCVA significantly improved from 0.27 ± 0.51 logMAR preoperatively to 0.17 ± 0.39 logMAR at the first postoperative month (*p* < 0.001). The degree of BCVA improvement was not associated with the PACD subtypes (*p* = 0.873) or the evidence of zonulopathy (*p* = 0.544). Intraoperative and postoperative adverse events are presented in Table 4. Among the 59 eyes with zonulopathy, 23 eyes required intraoperative use of CTR (39.0%, vs. no eye without zonulopathy; *p* < 0.001), 6 eyes had intraoperative evidence of vitreous prolapse and underwent anterior vitrectomy (10.2%, vs. no eye without zonulopathy; *p* < 0.001), and two eyes experienced posterior capsular rupture (3.4%. versus no eye without zonulopathy; *p* = 0.004) one of which required a scleral‐fixated IOL. Postoperatively, IOL decentration and anterior capsular phimosis were each observed among two zonulopathy eyes (3.4%, versus no eye without zonulopathy; *p* = 0.004). Early IOP spikes (first two postoperative months) occurred among 49 eyes of the cohort (6.1%) – 5 with and 44 without zonulopathy – without any differences between the two groups (*p* = 0.387). The causes of IOP spike in the zonulopathy group included vitreous prolapse requiring anterior vitrectomy in two eyes and postoperative ocular inflammation in the remaining three eyes. The causes of IOP spike among the eyes without zonulopathy included 20 eyes with mild ocular inflammation; six eyes with steroid response; two eyes with poor medication compliance; two eyes with PAS formation; one eye each with uveitis, tight scleral flap managed by laser suture lysis, hyphema, toxic anterior segment syndrome; and 10 eyes with an unclear cause. Among the 49 eyes with IOP spike, 23 underwent anterior chamber tap which constituted a significantly larger proportion of zonulopathy eyes (5 eyes; 8.5%) compared to those without zonulopathy (18 eyes; 2.4%; *p* = 0.008).

**TABLE 4:** Intraoperative and postoperative adverse events

Variable	Whole‐Cohort *N* = 806 *N* (%)	Eyes with zonulopathy *N* = 59 *N* (%)	Eyes without zonulopathy *N* = 747 *N* (%)	*p*‐value
Intraocular pressure spike	49 (6.1%)	5 (8.5%)	44 (5.9%)	0.387
Posterior capsular opacification within 1 year	44 (5.5%)	3 (5.1%)	41 (5.5%)	0.870
Intraoperative capsular tension ring use	23 (2.9%)	23 (39.0%)	0 (0.0%)	<0.001^a^
Anterior chamber tap	23 (2.9%)	5 (8.5%)	18 (2.4%)	0.008^a^
Anterior uveitis	17 (2.1%)	0 (0.0%)	17 (2.3%)	0.351
Postoperative peripheral anterior synechiae	10 (1.2%)	0 (0.0%)	10 (1.3%)	0.394
Vitreous prolapse requiring anterior vitrectomy	6 (0.7%)	6 (10.2%)	0 (0.0%)	<0.001^a^
Intraocular lens decentration	2 (0.2%)	2 (3.4%)	0 (0.0%)	0.004^a^
Anterior capsular phimosis	2 (0.2%)	2 (3.4%)	0 (0.0%)	0.004^a^
Posterior capsular rupture	2 (0.2%)	2 (3.4%)	0 (0.0%)	0.004^a^
Scleral‐fixated intraocular lens	1 (0.1%)	1 (1.7%)	0 (0.0%)	0.063
Posterior capsular wrinkling	1 (0.1%)	1 (1.7%)	0 (0.0%)	0.063
Pars‐plana posterior vitrectomy	1 (0.1%)	1 (1.7%)	0 (0.0%)	0.063
Retained cortical material	1 (0.1%)	1 (1.7%)	0 (0.0%)	0.063
Uveitis	1 (0.1%)	0 (0.0%)	1 (0.1%)	0.351
Hyphema	1 (0.1%)	0 (0.0%)	1 (0.1%)	0.351
Corneal endothelial decompensation	1 (0.1%)	0 (0.0%)	1 (0.1%)	0.351
Toxic anterior segment syndrome	1 (0.1%)	0 (0.0%)	1 (0.1%)	0.351

^a^ Denotes statistical significance.

Adverse events among eyes with zonulopathy were stratified and compared according to the intraoperative use of CTR (Table 4). None of the adverse events were found to be significantly more prevalent in one subgroup over another; however, CTR was not used in 5 out of 6 eyes with intraoperative vitreous prolapse, the two eyes with capsular phimosis, the two eyes with postoperative evidence of lens decentration, and the two eyes with posterior capsular rupture.

We additionally stratified the adverse events according to the PACD subtypes (Table 5); however, we did not find any significant differences in the rate of each adverse event between PACS, PAC and PACG eyes.

**TABLE 5:** Intraoperative and postoperative adverse events, stratified by angle closure diagnosis

Variable	Whole‐Cohort *N* = 806 *N* (%)	PACS *N* = 170 *N* (%)	PAC *N* = 349 *N* (%)	PACG *N* = 287 *N* (%)	*p*‐value
Intraocular pressure spike	49 (6.1%)	9 (5.3%)	25 (7.2%)	15 (5.2%)	0.566
Posterior capsular opacification within 1 year	44 (5.5%)	14 (8.2%)	15 (4.3%)	15 (5.2%)	0.179
Intraoperative capsular tension ring use	23 (2.9%)	5 (2.9%)	9 (2.6%)	9 (3.1%)	0.892
Anterior chamber tap	23 (2.9%)	4 (2.4%)	11 (3.2%)	8 (2.8%)	0.927
Anterior uveitis	17 (2.1%)	3 (1.8%)	10 (2.9%)	4 (1.4%)	0.434
Postoperative peripheral anterior synechiae	10 (1.2%)	0 (0.0%)	7 (2.0%)	3 (1.0%)	0.074
Vitreous prolapse requiring anterior vitrectomy	6 (0.7%)	1 (0.6%)	1 (0.3%)	4 (1.4%)	0.271
Intraocular lens decentration	2 (0.2%)	0 (0.0%)	0 (0.0%)	2 (0.7%)	0.171
Anterior capsular phimosis	2 (0.2%)	0 (0.0%)	0 (0.0%)	2 (0.7%)	0.171
Posterior capsular rupture	2 (0.2%)	0 (0.0%)	0 (0.0%)	2 (0.7%)	0.171
Scleral‐fixated intraocular lens	1 (0.1%)	0 (0.0%)	1 (0.3%)	0 (0.0%)	1.000
Posterior capsular wrinkling	1 (0.1%)	0 (0.0%)	0 (0.0%)	1 (0.3%)	0.567
Pars‐plana posterior vitrectomy	1 (0.1%)	0 (0.0%)	1 (0.3%)	0 (0.0%)	1.000
Retained cortical material	1 (0.1%)	0 (0.0%)	0 (0.0%)	1 (0.3%)	0.567
Uveitis	1 (0.1%)	0 (0.0%)	0 (0.0%)	1 (0.3%)	0.567
Hyphema	1 (0.1%)	0 (0.0%)	0 (0.0%)	1 (0.3%)	0.567
Corneal endothelial decompensation	1 (0.1%)	0 (0.0%)	1 (0.3%)	0 (0.0%)	1.000
Toxic anterior segment syndrome	1 (0.1%)	0 (0.0%)	1 (0.3%)	0 (0.0%)	1.000

Abbreviation: PACS: primary angle closure suspect; PAC: primary angle closure; PACG: primary angle closure glaucoma.

## 4 DISCUSSION

A thorough preoperative examination and identification of the physiological and anatomical variations are the key to success in cataract surgery. Zonular weakness is an example of such variations, and its under‐recognition can lead to detrimental intraoperative and postoperative complications. The prevalence of zonular weakness is reported among certain demographics, such as pseudoexfoliation syndrome and retinitis pigmentosa, with rates up to 13.1%^24^ and 18.8%,^25^ respectively; however, data regarding its prevalence in PACD remains scarce. The present study reports the prevalence of zonulopathy among a large cohort of PACD eyes with the hope of earlier identification of such cases and minimising the potential intraoperative and postoperative complications.

The results of our study evidenced a high prevalence of zonulopathy among PACD eyes, compared to the general population. In our cohort of 806 eyes with different types of PACD, 59 eyes had evidence of zonular weakness – representing 7.3% of the cohort. This contrasts with the prior data on the prevalence of zonulopathy in the general population ranging from 0.46% to 2.6%.^20^, ^21^, ^22^, ^23^ Of note, the majority of the previous studies included patients with known risk factors for zonulopathy such as history of posterior vitrectomy; whereas in the present study, we excluded these eyes to minimise potential confounding factors. Thus, it can be postulated that the rate of zonulopathy in our population could only be an under‐estimation, and the true prevalence of this pathology among PACD eyes is perhaps higher. Another point of consideration is that prior studies reported the prevalence of zonulopathy in the general population according to the number of eyes (including bilateral eyes of eligible patients).^20^, ^21^, ^22^, ^23^ To remain consistent with the literature and for comparative purposes, we kept our reporting technique the same and described the prevalence according to the number of eligible eyes.

The higher prevalence of zonulopathy in PACD compared to the general population, evidenced in this large cohort, suggests that zonulopathy could indeed be an under‐recognised mechanism for PACD. More precisely, we hypothesize that zonular weakness can allow forward movement of the lens leading to iridotrabecular contact and closure of the anterior chamber angle. Our hypothesis is corroborated by a previous case–control study reporting higher rates of zonular instability among eyes with a history of acute angle closure attack.^17^ An alternative explanation for this association is that zonular laxity leads to a suboptimal tension on the lens in the equatorial plane that results in a more spherical lens, a shallower anterior chamber, and perhaps angle closure.

Unlike primary pigment dispersion syndrome which most commonly occurs bilaterally in anatomically predisposed eyes, SPDS is thought to be unilateral and acquired through trauma, surgery, intraocular tumours or reverse‐pupillary block due to elevated IOP.^26^ To our knowledge, a possible association between PACD and SPDS has not been previously investigated. Here, we evidenced SPDS among 17.5% of the PACD eyes. Zonular weakness can lead to increased anteroposterior lens thickness along with anterior pushing of the lens, both of which can promote rubbing of posterior iris against the lens or zonules, causing pigment release. Based on the high prevalence of SPDS among our PACD population, it may be warranted in future revisions of the classification of angle closure disease to specify whether SPDS is present, as it can be an important clinical sign of possible intraoperative zonular instability, and the patients with heavier trabecular pigmentation may benefit from additional angle procedures such as trabecular removal or the insertion of stents.

Stratification of PACD into PACS, PAC and PACG did not yield any significant inter‐group differences with regard to the prevalence of zonulopathy. Given that PACD is a spectrum progressing from PACS with normal IOP to PAC and PACG with elevated IOP,^1^ the absence of difference between the rate of zonulopathy in PACS, PAC, and PACG further supports our hypothesis regarding the contributory role of zonulopathy in the development of PACD as opposed to other mechanisms such as elevated IOP.

Zonulopathy has been associated with a variety of intraoperative and postoperative adverse events including vitreous prolapse, capsular rupture, capsular contraction or phimosis, IOL dislocation or decentration, and pseudophacodonesis.^10^, ^11^, ^12^ In our study, a number of clinically and statistically significant adverse events were noted among eyes with zonulopathy compared to those without. Intraoperatively, vitreous prolapse – which is not uncommon among eyes with zonular deficiency – was observed among 10.2% of zonulopathy eyes. Two zonulopathy eyes (3.4%) experienced posterior capsular rupture, one of which required implantation of a scleral‐fixated IOL. Other adverse events observed in zonulopathy eyes were postoperative IOL decentration (3.4%), and anterior capsular phimosis (3.4%) – neither of which was observed in eyes without zonulopathy. These phenomena can negatively affect the visual outcomes and, in some cases, may require reoperation.

In eyes with evidence of zonular instability, the intraoperative use of CTR can help prevent intraoperative and postoperative complications.^27^ CTR serves to maintain the circular contour of the capsule while distributing the intraoperative forces equally over zonules to minimise drastic and disproportionate force to areas with zonular weakness.^27^ In our cohort, CTR was used in 23 eyes with zonulopathy (39.0% of zonulopathy eyes). Stratification of adverse events based on intraoperative CTR use revealed that CTR was not used in five of the six eyes with intraoperative vitreous prolapse, the two eyes with capsular phimosis, the two eyes with postoperative evidence of lens decentration, and the two eyes which experienced intraoperative posterior capsular rupture. Although these differences were not statistically significant – perhaps due to the small number of events and sample size within this subpopulation – the clinical significance of the adverse events may justify a more liberal use of CTR in PACD eyes suspected of having zonulopathy.

As it stands, the absence of practical and universally established criteria to objectively assess the strength of the zonules makes the preoperative diagnosis of this pathology rather challenging. Imaging techniques such as ultrasound biomicroscopy (UBM) allow assessing the extent of zonular disruption and help with preoperative planning.^28^ Nonetheless, UBM remains imperfect, as it could miss subtle zonular abnormalities.^28^ Rapid advancements in technology and increased availability and implication of new imaging modalities can aid with earlier diagnosis of PACD eyes with zonular weakness which could have otherwise gone undiagnosed during the preoperative examination.

There are some limitations to our study that should be discussed. The present work is a case series of consecutive PACD eyes that underwent cataract surgery or CLE with or without concomitant glaucoma surgeries by a single surgeon and at a single ophthalmology centre. Thus, our findings may not necessarily be generalizable to certain populations or demographics. On the positive side, the single‐surgeon nature of the study limits the inter‐surgeon variability with regard to surgical technique or clinical examination. The absence of a second arm including open‐angle eyes limits our ability to compare the findings to a control group. However, to rectify this shortcoming, we compared the prevalence of zonulopathy in our cohort to that of the general population described by previous studies. While the possibility of registry or memory bias cannot be ruled out due to the retrospective nature of the study, we anticipate that this limitation could have only under‐estimated the prevalence of zonulopathy and its associated adverse events. Furthermore, the absence of long‐term follow‐up for some patients could have likely contributed to under‐estimation of the associated long‐term adverse events.

In summary, the present study is the first to report the prevalence of zonulopathy in a large cohort of PACD eyes. In this sizable sample of 806 consecutive PACD eyes, zonulopathy was evidenced among 7.3% of the eyes – a prevalence that is significantly higher than the general population. Also, SPDS was observed among 17.5% of the eyes. Based on this evidence, we suggest zonulopathy as a possible under‐recognised cause of PACD by allowing forward movement of the lens and narrowing the anterior chamber angle. The significant intraoperative and postoperative complications associated with zonulopathy can be minimised or prevented by its preoperative and early intraoperative recognition. To that end, we hope the findings of the present study inform ophthalmologists about the high prevalence of zonulopathy in PACD, and contribute to improving patient care.

## CONFLICT OF INTEREST

Paul Harasymowycz is a consultant for Allergan, Alcon, Glaukos, Ivantis, J and J Vision, Novartis, Santen, and Bausch and Lomb. Ali Salimi, Anthony Fanous, Harrison Watt, Mohamed Abu‐Nada, and Anna Wang have no disclosures.

## References

[1] Prum BE Jr , Herndon LW Jr , Moroi SE , et al. Primary angle closure preferred practice pattern([R]) guidelines. Ophthalmology. 2016;123(1):1‐P40. 10.1016/j.ophtha.2015.10.049 26581557

[2] Friedman DS , Foster PJ , Aung T , He M . Angle closure and angle closure glaucoma: what we are doing now and what we will be doing in the future. Clin Experiment Ophthalmol. 2012;40(4):381‐387. 22356460 10.1111/j.1442-9071.2012.02774.x

[3] Sun X , Dai Y , Chen Y , et al. Primary angle closure glaucoma: what we know and what we don't know. Prog Retin Eye Res. 2017;57:26‐45. 28039061 10.1016/j.preteyeres.2016.12.003

[4] Jonas JB , Aung T , Bourne RR , Bron AM , Ritch R , Panda‐Jonas S . Glaucoma. Lancet. 2017;390(10108):2183‐2193. 28577860 10.1016/S0140-6736(17)31469-1

[5] Vajaranant TS , Nayak S , Wilensky JT , Joslin CE . Gender and glaucoma: what we know and what we need to know. Curr Opin Ophthalmol. 2010;21(2):91‐99. 20051857 10.1097/ICU.0b013e3283360b7e PMC4326058

[6] Shen L , Melles RB , Metlapally R , et al. The Association of Refractive Error with glaucoma in a multiethnic population. Ophthalmology. 2016;123(1):92‐101. 26260281 10.1016/j.ophtha.2015.07.002 PMC4695304

[7] Radhakrishnan S , Chen PP , Junk AK , Nouri‐Mahdavi K , Chen TC . Laser peripheral Iridotomy in primary angle closure: a report by the American Academy of Ophthalmology. Ophthalmology. 2018;125(7):1110‐1120. 29482864 10.1016/j.ophtha.2018.01.015

[8] Baskaran M , Yang E , Trikha S , et al. Residual angle closure one year after laser peripheral Iridotomy in primary angle closure suspects. Am J Ophthalmol. 2017;183:111‐117. 28887116 10.1016/j.ajo.2017.08.016

[9] Azuara‐Blanco A , Burr J , Ramsay C , et al. Effectiveness of early lens extraction for the treatment of primary angle‐closure glaucoma (EAGLE): a randomised controlled trial. Lancet. 2016;388(10052):1389‐1397. 27707497 10.1016/S0140-6736(16)30956-4

[10] Dureau P . Pathophysiology of zonular diseases. Curr Opin Ophthalmol. 2008;19(1):27‐30. 18090894 10.1097/ICU.0b013e3282f29f01

[11] Miyoshi T , Fujie S , Yoshida H , Iwamoto H , Tsukamoto H , Oshika T . Effects of capsular tension ring on surgical outcomes of premium intraocular lens in patients with suspected zonular weakness. PLoS One. 2020;15(2):e0228999. 32092103 10.1371/journal.pone.0228999 PMC7039513

[12] Shingleton BJ , Neo YN , Cvintal V , Shaikh AM , Liberman P , O'Donoghue MW . Outcome of phacoemulsification and intraocular lens implantion in eyes with pseudoexfoliation and weak zonules. Acta Ophthalmol. 2017;95(2):182‐187. 27230126 10.1111/aos.13110

[13] Jehan FS , Mamalis N , Crandall AS . Spontaneous late dislocation of intraocular lens within the capsular bag in pseudoexfoliation patients. Ophthalmology. 2001;108(10):1727‐1731. 11581041 10.1016/s0161-6420(01)00710-2

[14] Yasuda A , Ohkoshi K , Orihara Y , Kusano Y , Sakuma A , Yamaguchi T . Spontaneous luxation of encapsulated intraocular lens onto the retina after a triple procedure of vitrectomy, phacoemulsification, and intraocular lens implantation. Am J Ophthalmol. 2000;130(6):836‐837. 11124310 10.1016/s0002-9394(00)00630-9

[15] Shingleton BJ , Crandall AS , Ahmed II . Pseudoexfoliation and the cataract surgeon: preoperative, intraoperative, and postoperative issues related to intraocular pressure, cataract, and intraocular lenses. J Cataract Refract Surg. 2009;35(6):1101‐1120. 19465298 10.1016/j.jcrs.2009.03.011

[16] Tanner L , Gazzard G , Nolan WP , Foster PJ . Has the EAGLE landed for the use of clear lens extraction in angle closure glaucoma? And how should primary angle closure suspects be treated? Eye (Lond). 2020;34(1):40‐50. 31649349 10.1038/s41433-019-0634-5 PMC7002615

[17] Kwon J , Sung KR . Factors associated with zonular instability during cataract surgery in eyes with acute angle closure attack. Am J Ophthalmol. 2017;183:118‐124. 28916480 10.1016/j.ajo.2017.09.003

[18] Salimi A , Winter A , Li C , Harasymowycz P , Saheb H . Effect of topical corticosteroids on early postoperative intraocular pressure following combined cataract and trabecular microbypass surgery. J Ocul Pharmacol Ther. 2019;35(7):413‐420. 31373857 10.1089/jop.2019.0019

[19] Salimi A , Nithianandan H , Al Farsi H , Harasymowycz P , Saheb H . Gonioscopy‐assisted transluminal Trabeculotomy in younger to middle‐aged adults: one‐year outcomes. Ophthalmol Glaucoma. 2021;4(2):162‐172. 32891748 10.1016/j.ogla.2020.08.014

[20] Soliman MK , Hardin JS , Jawed F , et al. A database study of visual outcomes and intraoperative complications of Postvitrectomy cataract surgery. Ophthalmology. 2018;125(11):1683‐1691. 30041814 10.1016/j.ophtha.2018.05.027

[21] Jaycock P , Johnston RL , Taylor H , et al. The cataract National Dataset electronic multi‐Centre audit of 55,567 operations: updating benchmark standards of care in the United Kingdom and internationally. Eye (Lond). 2009;23(1):38‐49. 18034196 10.1038/sj.eye.6703015

[22] Trikha S , Agrawal S , Saffari SE , Jayaswal R , Yang YF . Visual outcomes in patients with zonular dialysis following cataract surgery. Eye (Lond). 2016;30(10):1331‐1335. 27285326 10.1038/eye.2016.108 PMC5129851

[23] Girgis R , Salam T , Verma S . To assess the results of the clinical outcome of high volume cataract operations performed by a fellow and a consultant in Moorfields eye hospital. Eye (Lond). 2012;26(5):756‐757. 22302067 10.1038/eye.2011.357 PMC3351043

[24] Avramides S , Traianidis P , Sakkias G . Cataract surgery and lens implantation in eyes with exfoliation syndrome. J Cataract Refract Surg. 1997;23(4):583‐587. 9209997 10.1016/s0886-3350(97)80219-2

[25] Dikopf MS , Chow CC , Mieler WF , Tu EY . Cataract extraction outcomes and the prevalence of zonular insufficiency in retinitis pigmentosa. Am J Ophthalmol. 2013;156(1):82‐88. e82. 23628349 10.1016/j.ajo.2013.02.002

[26] Sivaraman KR , Patel CG , Vajaranant TS , Aref AA . Secondary pigmentary glaucoma in patients with underlying primary pigment dispersion syndrome. Clin Ophthalmol. 2013;7:561‐566. 23569351 10.2147/OPTH.S42456 PMC3615837

[27] Weber CH , Cionni RJ . All about capsular tension rings. Curr Opin Ophthalmol. 2015;26(1):10‐15. 25390861 10.1097/ICU.0000000000000118

[28] Pavlin CJ , Buys YM , Pathmanathan T . Imaging zonular abnormalities using ultrasound biomicroscopy. Arch Ophthalmol. 1998;116(7):854‐857. 9682697 10.1001/archopht.116.7.854

